# Hyperglycemia and hyperlipidemia blunts the Insulin-Inpp5f negative feedback loop in the diabetic heart

**DOI:** 10.1038/srep22068

**Published:** 2016-02-24

**Authors:** Danna Bai, Yajun Zhang, Mingzhi Shen, Yongfeng Sun, Qing Xia, Yingmei Zhang, Xuedong Liu, Haichang Wang, Lijun Yuan

**Affiliations:** 1 Department of Cardiology, Xijing Hospital, the Fourth Military Medical University, Xi’an 710032, China; 2 323 Hospital of PLA, Xi’an 710054, China; 3 Department of Ultrasound Diagnostics, Tangdu Hospital, the Fourth Military Medical University, Xi’an 710038, China; 4 Department of Cardiology, Hainan Branch of PLA General Hospital, Sanya 572013, China; 5 Department of Neurology, Xijing Hospital, the Fourth Military Medical University, Xi’an 710032, China

## Abstract

The leading cause of death in diabetic patients is diabetic cardiomyopathy, in which alteration of Akt signal plays an important role. Inpp5f is recently found to be a negative regulator of Akt signaling, while its expression and function in diabetic heart is largely unknown. In this study, we found that in both the streptozotocin (STZ) and high fat diet (HFD) induced diabetic mouse models, Inpp5f expression was coordinately regulated by insulin, blood glucose and lipid levels. Increased Inpp5f was inversely correlated with the cardiac function. Further studies revealed that Insulin transcriptionally activated Inpp5f in an Sp1 dependent manner, and increased Inpp5f in turn reduced the phosphorylation of Akt, forming a negative feedback loop. The negative feedback plays a protective role under diabetic condition. However, high blood glucose and lipid, which are characteristics of uncontrolled diabetes and type 2 diabetes, increased Inpp5f expression through activation of NF-κB, blunts the protective feedback. Thus, our study has revealed that Inpp5f provides as a negative feedback regulator of insulin signaling and downregulation of Inpp5f in diabetes is cardioprotective. Increased Inpp5f by hyperglycemia and hyperlipidemia is an important mediator of diabetic cardiomyopathy and is a promising therapeutic target for the disease.

The incidence of diabetes mellitus increases dramatically over time and has become one of the world’s main disablers and killers. It is estimated that the number of diabetic patients would increase to at least 300 million by 2025, according to the WHO^1^. Diabetic cardiomyopathy, namely ventricular dysfunction independent of coronary artery disease or hypertension, is one of the leading causes of morbidity and mortality in these diabetic individuals^2^,^3^. Insulin deficiency in Type 1 diabetes and insulin resistance in Type 2 diabetes, together with the common metabolic stress (mainly hyperglycemia and hyperlipidemia), are well-established risk factors for diabetic cardiomyopathy^4^,^5^. These risk factors together lead to decreased glucose uptake/oxidation and increased fatty acids uptake/oxidation. This metabolic shift plays an essential role in the establishment of cardiomyopathy despite the detailed molecular processes are not fully understood yet.

Insulin/PI3K/Akt signaling pathway centers in the whole molecular events, linking the extracellular cues in diabetes and cardiac cell response together^6^. It is thus interesting to explore how the Insulin/PI3K/Akt pathway is fine-tuned and how they go awry in the context of diabetes. Recently, maladaptive immune response and inflammation has been found to involve in the cardiovascular resistance^7^. Activation of NF-κB has been found in multiple cells in the context of diabetes, such as vascular smooth muscle cells, cardiomyocytes^8^,^9^. Cardiac specific inhibition of NF-κB significantly improves the cardiac function^10^, further indicating that inflammation might be a key cause of diabetic cardiomyopathy. However, how inflammation links to the cardiac dysfunction, especially, how inflammation links to the PI3K/AKT aberration, is totally unknown. PI3K/AKT signal pathway is negatively regulated by multiple phosphatases, such as PTEN, one of the intensively studied phosphatases. Increased PTEN activity has been suggested in diabetic cardiomyopathy^11^,^12^. Similar as PTEN, another phosphatase named as PHLPP (Pleckstrin homology domain leucine-rich repeat protein phosphatase) is also considered as a bigger player in diabetes^13^. Besides PTEN and PHLPP, Inositol polyphosphate-5-phosphatase (Inpp5f) is identified and characterized as a new member of polyphosphoinositide phosphatases. Accumulating evidence demonstrates that Inpp5f can degrade both phosphatidylinositol 4,5-bisphosphate (PtdIns[4,5]P_2_ [PIP2]) and phosphatidylinositol 3,4,5-trisphosphate (PtdIns[3,4,5]P_3_ [PIP3]) by removing the 5′phosphate from the inositol ring and thus inhibit Akt signaling^14^,^15^,^16^,^17^. The study by Zhu W *et al*. has revealed that Inpp5f knockout mice are sensitive to hypertrophy, while Inpp5f transgenic mice are resistant to hypertrophy^18^. All of these data suggest that Inpp5f is a functionally important endogenous modulator of Akt signal and might plays an important role in response to physiological and pathological stimuli. To our knowledge, whether Inpp5f plays a role in the cardiomyopathy via fine-tuning Insulin/PI3K/PKB/Akt pathway in the context of diabetes has not been determined.

In this study, we examined the expression of Inpp5f in both STZ- and HFD-induced diabetes mouse model and correlated its expression levels with the blood chemistry parameters and cardiac functional parameters. By using the cell culture model, we explored the mechanism how Inpp5f regulates the Insulin/PI3K/PKB/Akt pathway and thus glucose uptake in the context of diabetes. Our study has revealed that Inpp5f is a downstream target of insulin signaling. Down-regulation of Inpp5f in response of decreased insulin signaling activity rescues Akt activity in a negative feedback manner and is cardioprotective in the context of diabetes, while uncontrolled hyperglycemia and hyperlipidemia blocks the protective feedback through upregulating Inpp5f.

## Results

### Increased Inpp5f expression in diabetic hearts correlates with metabolic stress

**Figure 1:**
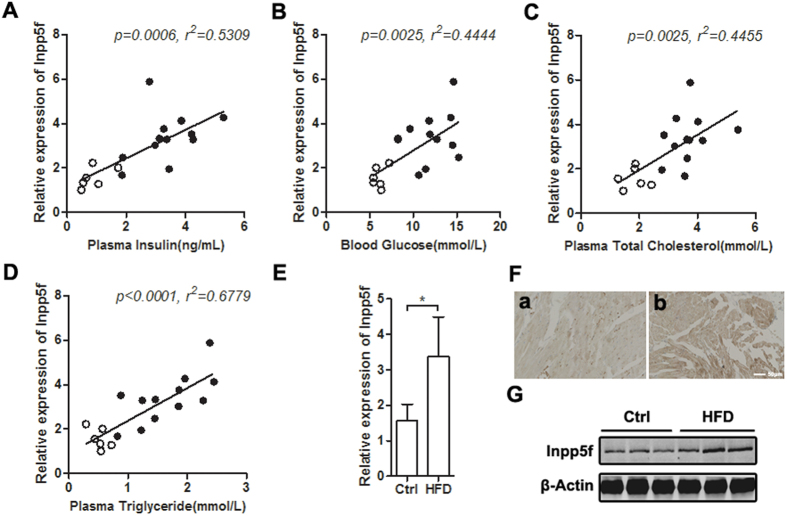
Cardiac expression of Inpp5f in HFD induced diabetic mice. (**A–D**) Correlation of cardiac Inpp5f expression and insulin (**A**), blood glucose (**B**), Cholesterol (**C**) and Triglyceride (**D**) in mice with Chow and HFD fed for 26 weeks. The lowest expression of Inpp5f was set as 1. (**E**) Cardiac mRNA expression of Inpp5f in the above samples. *P < 0.05 versus respective control group. (**F**) Immunohistochemistry staining of Inpp5f in the hearts from control and HFD fed mice. Data are representative of three chow and HFD fed mice. (**G**) Protein expression of Inpp5f in above samples. Data presented are representative of 6 Chow and 12 HFD fed mice.

We examined the expression of Inpp5f in two established experimental models of diabetes: Type 1 induced by STZ and Type 2 induced by high-fat diet (HFD). As expected, STZ model displayed decreased insulin while HFD model showed increased insulin (Supplementary Fig. S1A, and Fig. 1A). Both models manifested increases in nonfasting serum glucose, cholesterol and triglyceride (Supplementary Fig. S1B–D and Fig. 1B–D). Increase of Inpp5f expression at both mRNA and protein levels were observed in HFD induced diabetes model (Fig. 1A,G). Immunohistochemistry analysis further confirmed the increase of Inpp5f in the heart of HFD mice (Fig. 1F). Notably, cardiac Inpp5f expression in the HFD mice varied in a wild range as seen from the huge error bar (Fig. 1E), suggesting that Inpp5f expression might be affected by multiple factors. Correlation study further revealed that Inpp5f expression was linearly correlated with the blood insulin, glucose and lipid levels (Fig. 1A–D). Different from the HFD model, Inpp5f expression in the STZ-induced diabetic model was more complicated. Inpp5f expression decreased in the first 15–45 days after STZ induction, and was restored gradually afterwards (Supplementary Fig. S1E–G). Apparently, the dynamics of Inpp5f during the diabetes development in the STZ model did not correlate with the insulin, glucose and lipid levels. However, the decrease of Inpp5f in the first 15–45 days and restoration 60 days after STZ induction coincided with the decrease of insulin and increase of metabolic stress respectively (Supplementary Fig. S1A–D).

### Increased Inpp5f expression in diabetic hearts adversely relates with cardiac function

**Figure 2:**
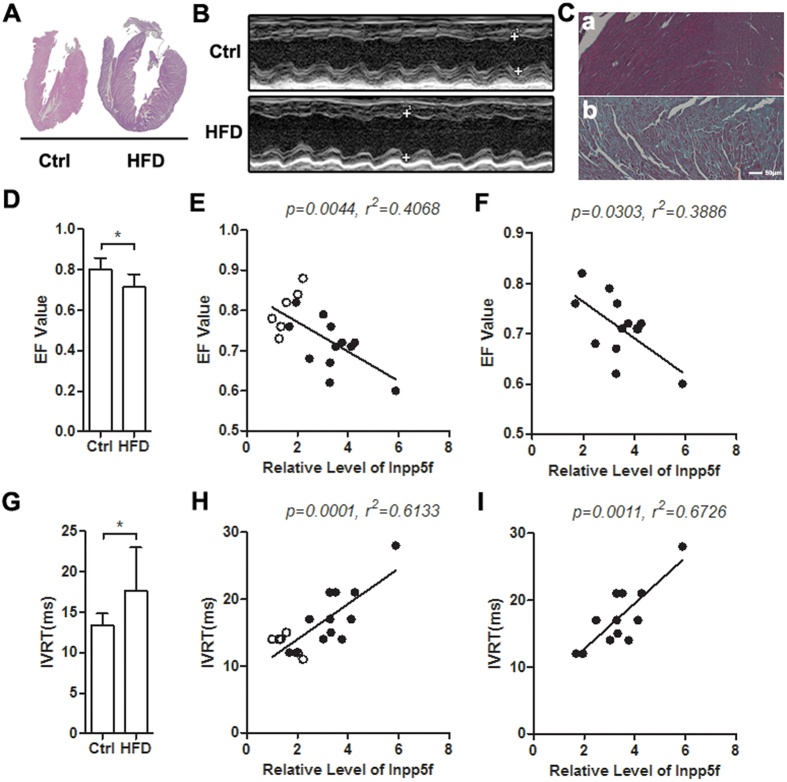
Increased Inpp5f correlates with decreased cardiac function in HFD fed mice. (**A**) HE staining of the hearts from Chow and HFD fed mice. Data presented are a representative of 6 Chow and 12 HFD fed mice. (**B**) M-mode echocardiography of the above Chow and HFD fed mice before sacrifice. (**C**) Masson Trichrome staining of the hearts from Chow (a) and HFD (b) fed mice. Data presented are a representative of 6 Chow and 12 HFD fed mice. (**D**) Ejection fraction (EF) value of cardiac systolic function in both Ctrl and HFD fed mice. *P < 0.05, n = 6 for Chow and n = 12 for HFD group. (**E**) Correlation of Inpp5f expression and EF value in all the 6 control and 12 HFD mice. The lowest expression of Inpp5f was set as 1. (**F**) Correlation of Inpp5f expression and EF value in the 12 HFD mice only. (**G**) Isovolumic relaxation time (IVRT) of cardiac diastolic function in the same Ctrl and HFD fed mice as in (**D**). *P < 0.05, n = 6 in each group. (**H**) Correlation of Inpp5f expression and IVRT value in all the 6 control and 12 HFD mice. *P < 0.05. (**I**) Correlation of Inpp5f expression and IVRT value in 12 HFD diabetic mice only. *P < 0.05.

Next, we explored the correlation between Inpp5f expression and cardiac function. Generally, diabetic mice from both models manifested marked myocardial dysfunction, exemplified by both hypertrophy (Supplementary Fig. S2A,B, Fig. 2A,B), obvious fibrosis (Supplementary Fig. S2C, Fig. 2C) in histology, decreased ejection fraction (EF) value and increased isovolumetric relaxation time (IVRT) by echocardiography (Supplementary Fig. S2D–I and Fig. 2D–I). Correlation analysis revealed that Inpp5f expression negatively correlated with EF value, while positively correlated with the IVRT (Fig. 2E,F,H,I), in all the control and HFD mice. In the STZ model, it was found that Inpp5f expression was weakly correlated with the cardiac function when both the control and diabetic mice were considered (Supplementary Fig. S2E,H). However, Inpp5f expression and cardiac function was linearly related when diabetic samples only were included (Supplementary Fig. S2F,I). All of the above data indicate that increase of Inpp5f in both diabetes models negatively correlates with the decrease of cardiac function.

### Insulin, high glucose and FFA coordinately regulate Inpp5f expression

**Figure 3:**
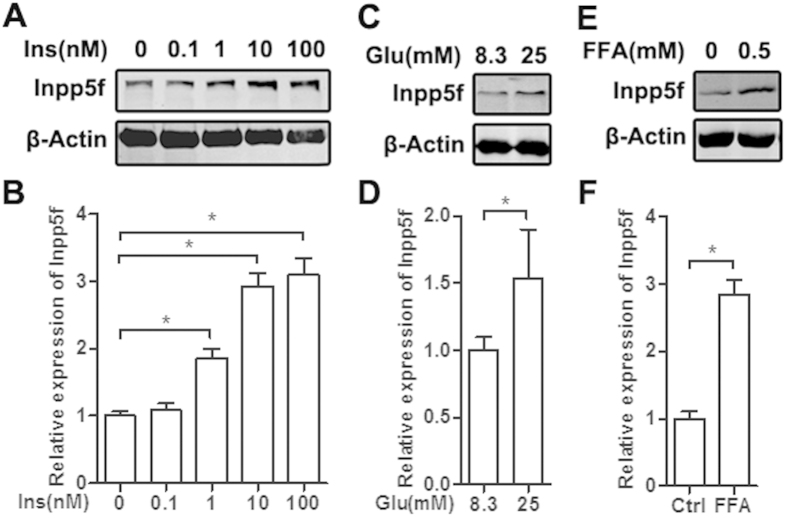
Insulin, high glucose and FFA increase Inpp5f expression. (**A**) Serum starved H9C2 cells were treated with insulin at the indicated concentrations, and Western blot assay revealed a dose dependent upregulation of Inpp5f at protein level. Data presented are representative of triplicates. (**B**) qPCR analysis of Inpp5f mRNA in the above samples. (**C**) Serum starved H9C2 cells were grown in the low glucose medium 24 hrs before glucose treatment. Inpp5f expression at protein level was tested by Western blot. n = 3. (**D**) qPCR analysis of Inpp5f mRNA in the above samples. (**E**) Serum starved H9C2 cells were grown in the low glucose medium 24 hrs before free fatty acids treatment. Inpp5f expression at protein level was tested by Western blot. n = 3. (**F**) qPCR analysis of Inpp5f mRNA in the above samples.

From the above data, we assumed that insulin, high glucose and FFA might be involved in the expression regulation of Inpp5f in the diabetic heart. Treatment of insulin in serum starved H9C2 cells dose dependently increased Inpp5f expression at both protein and mRNA levels (Fig. 3A,B). In addition, both high glucose and FFA increased Inpp5f expression at protein and mRNA levels (Fig. 3C–F). Coordinate regulation of Inpp5f by insulin, high glucose and FFA well explain the observed expression profile of Inpp5f in both STZ and HFD induced diabetic hearts.

### Hyperglycemia and hyperlipidemia blocks the Insulin-Inpp5f negative feedback loop

**Figure 4:**
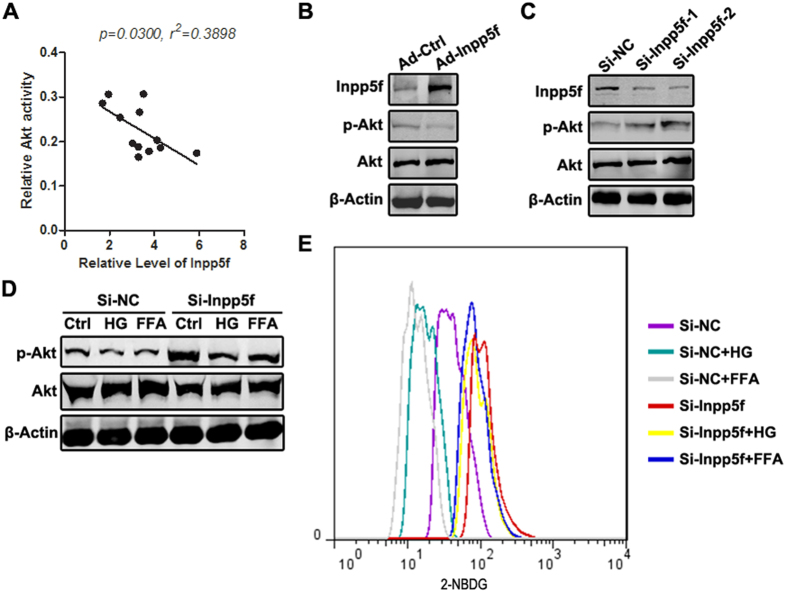
Hyperglycemia and hyperlipidemia blocks the Insulin-Inpp5f negative feedback loop. (**A**) Correlation between Inpp5f expression and Akt activity in HFD induced diabetic hearts. n = 12 (**B**) Overexpression of Inpp5f reduced Akt phosphorylation. H9C2 cells were infected with control and Inpp5f overexpressing adenovirus. Inpp5f, Akt, phosphorylated Akt and β-actin expression were analyzed by Western blot. Representative data of triplicates. (**C**) Knockdown of Inpp5f increased Akt phosphorylation. H9C2 cells were transfected with scramble siRNA or siRNA against Inpp5f. Inpp5f, Akt, phosphorylated Akt and β-actin expression were analyzed by Western blot. Representative data of triplicates. (**D**) H9C2 cells transfected with scramble siRNA or siRNA were conditioned with serum free, high glucose and FFA medium before Insulin treatment. Phosphorylated Akt and total Akt were detected by Western blot. (**E**) Knockdown of Inpp5f rescued the glucose/FFA mediated inhibition of 2-NBDG uptake. H9C2 cells transfected with scramble siRNA or siRNA were conditioned with serum free, high glucose and FFA medium before Insulin treatment. Fifteen minutes later, 2-NBDG uptake was monitored by flow cytometry.

To further explore the effects of altered Inpp5f in the context of diabetes, we mainly focused on Akt activity and glucose uptake, the key molecular and cellular readouts of insulin signaling. Cardiac Inpp5f expression adversely correlated well with the Akt activity in the HFD mice and STZ mice models (Fig. 4A, Supplementary Fig. S3), while no correlation was observed when the control mice were included. In other words, Inpp5f is a potent regulator of Akt activity in the context of diabetes. Consistent with previous study^18^, overexpression of Inpp5f in H9C2 cells reduced Akt phosphorylation, while knockdown of Inpp5f increased Akt phosphorylation (Fig. 4B,C). In the cell model, high glucose and FFA pretreatment reduced Akt phosphorylation and 2-NBDG uptake, which was greatly rescued by Inpp5f knockdown (Fig. 4D,E). These data suggest that, increase of Inpp5f might be one of the key mediators of metabolic stress (hyperglycemia and hyperlipidemia) induced insulin signaling deficiency.

To further test the role of increased Inpp5f in diabetic heart, we tried to knockdown of Inpp5f in the heart by gold nanoparticle conjugated siRNAs (AuNP-siRNA). As shown in supplementary Fig. S4, AuNP-siRNA delivered the siRNAs into the heart at a compromised proportion. However, we did not observe any obvious rescue of the cardiac function, which might be explained by low transfection efficiency and short half-life of the siRNAs when compared with the sustained increase of Inpp5f. Future study using Inpp5f KO mice would be helpful to answer this question *in vivo*.

### Insulin and high glucose/FFA increase Inpp5f expression in Sp1 and NF-κB dependent manner respectively

**Figure 5:**
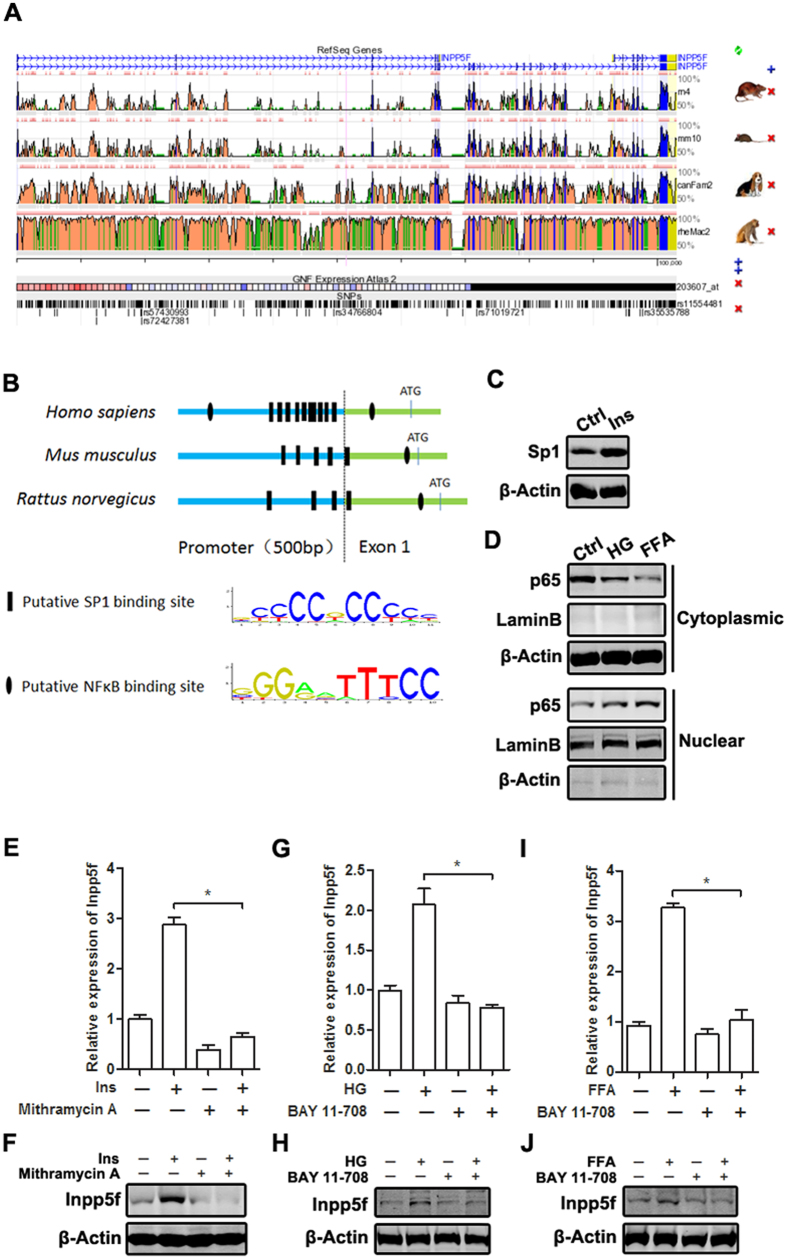
Insulin, high glucose and FFA increase Inpp5f expression by different transcription factors. (**A**) Comparison of the gene sequences of Inpp5f among different species using web-based ECR (see article in ref. 35). (**B**) The 500 bp promoter region and the first exon of Inpp5f in different species were screened for the putative Sp1 and NF-κB binding sites by JASPAR web (see article in ref. 36). (**C**) Serum starved H9C2 cells were treated with Insulin and expression of Sp1 was tested by Western blot. (**D**) H9C2 cells were treated with HG or FFA for 24 hrs, p65 nuclear translocation was analyzed by western blot in cytoplasmic and nuclear extract respectively. (**E,F**) H9C2 cells were treated with insulin, Sp1 inhibitor or the combination. Inpp5f expression at mRNA (**E**) and protein levels (**F**) was examined by qPCR and Western Blot. *P < 0.05, n = 3. (**G–H**) H9C2 cells were treated with HG, NF-κB inhibitor, or the combination. Inpp5f expression at mRNA (**G**) and protein levels (**H**) was examined by qPCR and Western Blot. *P < 0.05, n = 3. (**I**,**J**) H9C2 cells were treated with FFA, NF-κB inhibitor, or the combination. Inpp5f expression at mRNA (**I**) and protein levels (**J**) was examined by qPCR and Western Blot. *P < 0.05, n = 3.

To further clarify how insulin, high glucose and FFA increase Inpp5f expression, we analyzed the promoter sequences of Inpp5f among different species and explored the regulatory mechanism. Both the promoter region and coding region of Inpp5f are evolutionarily conserved among species (Fig. 5A), with the detailed alignment information shown in Supplementary Fig. S5. The identity between mouse and rat even reaches 87%. There were multiple conserved Sp1 and NF-κB binding sites in the 500 bp promoter region and the first exon of Inpp5f among human, mouse and rat (Fig. 5B and Supplementary Fig. S5). Previous studies have revealed that Sp1 is a mediator of insulin signaling^19^, while high glucose and FFA can activate NF-κB in diabetes^8^,^9^,^10^, raising the possibility that insulin and high glucose/FFA might induce Inpp5f expression in Sp1 and NF-κB dependent manner respectively. Consistently, we found that insulin increased endogenous expression of Sp1 (Fig. 5C), while high glucose and FFA induced nuclear translocation of p65 (Fig. 5D). Inhibition of Sp1 by mithramycin A reduced the basal and insulin induced Inpp5f expression at both mRNA and protein levels (Fig. 5E,F). Inhibition of NF-κB by Bay 11–708 also blocked high glucose and FFA induced Inpp5f, while had little effects on its basal expression (Fig. 5G–J).

**Figure 6:**
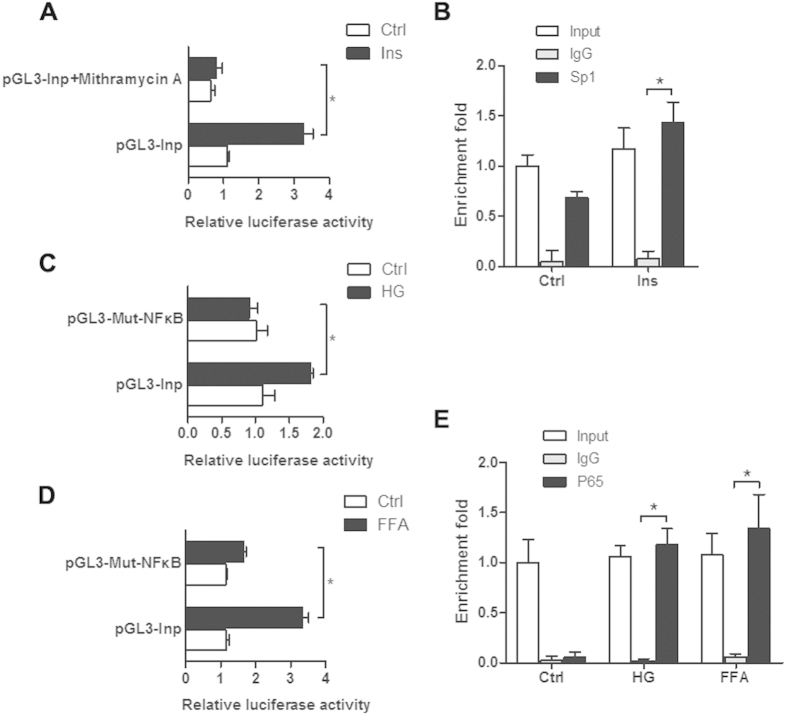
Sp1 and NF-κB transcriptionally activate Inpp5f expression. (**A**) H9C2 cells were co-transfected with mouse Inpp5f promoter reporter (pGL3-Inp) and pRL-TK and additionally treated with ctrl, insulin with/or Sp1 inhibitor. *P < 0.05, n = 3. (**B**) H9C2 cells treated with either ctrl or insulin were harvested for ChIP analysis by Sp1 antibody. (**C**) H9C2 cells were transfected with mouse pGL3-Inp or the NF-κB mutant counterpart together with pRL-TK. Cells were additionally treated with ctrl or HG 12 hours after transfection. *P < 0.05, n = 3. (**D**) H9C2 cells were transfected with mouse pGL3-Inp or the NF-κB mutant counterpart together with pRL-TK and additionally treated with ctrl or FFA. *P < 0.05, n = 3. (**E**) H9C2 cells with ctrl, HG or FFA treatment were harvested for ChIP analysis with p65 antibody.

To further confirm the role of Sp1 and NF-κB in the induction of Inpp5f under diabetes context, mouse Inpp5f regulatory region (−734 ~ +1, with the A in ATG designated as +1), which covers about 500 bp promoter region and part of the first exon, was cloned into pGL3 basic reporter vector. Reporter luciferase activity showed that insulin increased the promoter activity, which was blocked by Sp1 inhibitor (Fig. 6A). ChIP analysis using the primers flanking the promoter region −418 ~ −271 of mouse Inpp5f core promoter, which covers the region harboring conservative Sp1 binding sites across the species, further confirmed that Sp1 did bind to the core promoter. The interaction between Sp1 and Inpp5f promoter was further enhanced by insulin treatment (Fig. 6B). High glucose and FFA also increased the luciferase activity of wildtype reporter (Fig. 6C,D), while had no effects on the mutant reporter with the NF-κB binding site mutated (Fig. 6C,D). ChIP analysis using the primers flanking the NF-κB binding site (−88 ~ +29 of mouse Inpp5f promoter) revealed that abundant p65 bound to the corresponding exon region under high glucose and FFA treatment, while there was no significant binding at the basal condition (Fig. 6E).

**Figure 7:**
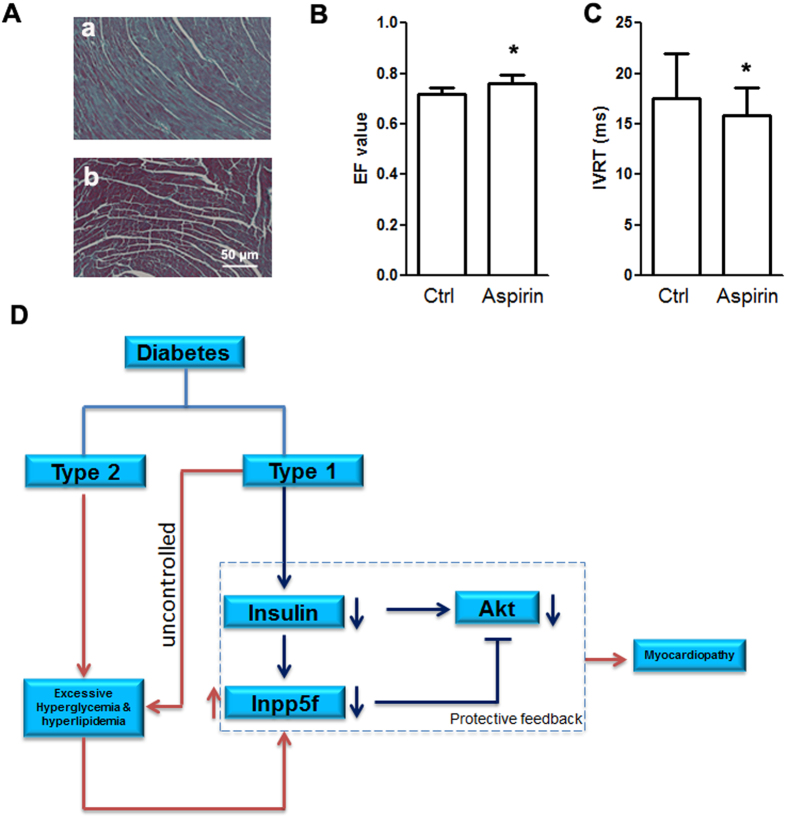
Working model of the role of Inpp5f in diabetic cardiomyopathy. (**A**) Masson Trichrome staining of the hearts from the HFD mice with either control (a) or aspirin (b) treatment. Data presented are a representative of 7 mice in each group. (**B**) Ejection fraction (EF) value of cardiac systolic function of HFD fed mice additionally treated with either control or aspirin. *P < 0.05, n = 7 for both groups. (**C**) Isovolumic relaxation time (IVRT) of cardiac diastolic function of the same mice as above. *P < 0.05. (**D**) In diabetes, insufficient insulin signals due to absolute deficiency of insulin (T1D) or insulin resistance (T2D) resulted in decrease of Inpp5f. Decreased Inpp5f in turn rescues Akt phosphorylation in a negative feedback manner. While hyperglycemia and hyperlipidemia in the advanced or uncontrolled diabetes increase Inpp5f and thus block the feedback, aggravating the cardiomyopathy.

Since NF-κB plays an essential role in hyperglycemia and hyperlipidemia induced Inpp5f, we next tested whether NF-κB inhibition could improve the cardiac function in HFD mice. HFD induced diabetic mice were randomly divided into 2 groups (n = 7 in each group) receiving either placebo or the NF-κB inhibitor aspirin (40 mg/L) in their drinking water since the 12th week of HFD induction. As expected, aspirin treatment greatly decreased the fibrosis (Fig. 7A) and rescued the cardiac function (Fig. 7B–C).

## Discussion

By using both STZ and HFD induced diabetic mouse model, we here for the first time found that Inpp5f is coordinately regulated by insulin, high glucose and free fatty acids in diabetic hearts. Higher expression of Inpp5f indicates worse cardiac function. Decreased insulin signaling in diabetic hearts reduces Inpp5f expression, which in turn at least partially rescues the phosphorylation and activity of Akt and glucose uptake, forming a cardio-protective: Insulin-Inpp5f-Akt negative feedback loop. In addition, high concentration of glucose and free fatty acid, which are two metabolic stress characteristics of diabetes, also increase Inpp5f expression in an NF-κB dependent manner. Induction of Inpp5f by the metabolic stress blunts the cardio-protective Insulin-Inpp5f-Akt feedback (Fig. 7D).

Diabetic cardiomyopathy is a kind of heart disease independent of hypertension or coronary atherosclerosis, and there is a large body of evidence implicates insulin deficiency/resistance in the pathogenesis of these disorders. Up to now how insulin signal is fine-tuned is poorly understood. The feedback regulatory mode is a common mechanism in the context of development and disease^20^. Therapeutically targeting the feedback loop is considered as a promising strategy for disease treatment. Up to now, there are few studies revealing the instinct feedback loops in the context of diabetes. Previously, FoxO proteins are established downstream targets of insulin signaling; at the same time, they mediate feedback control to govern critical aspects of the signaling cascade^21^,^22^,^23^. Recently, Pavan *et al*.^24^ revealed that activation of FoxO1 is an important mediator of diabetic cardiomyopathy and is a promising therapeutic target for the disease. Our study here established a new molecule, namely Inpp5f, function in a similar way as FOXOs. Therapeutically reducing Inpp5f expression or biologically inhibiting its activity would be a promising strategy in protecting cardiac function in the context of diabetes, especially under the context of hyperglycemia and hyperlipidemia.

Previous studies indicate both decrease and increase of Akt activity have been found in various tissues and cells in diabetes depending on experimental and clinical contexts^25^. In the context of type 2 diabetes, there are extensive data showing clear insulin resistance in skeletal muscle, as seen in less insulin stimulation of glucose uptake, oxidation, or activation of Akt. However, there is growing evidence that there is little or no loss of insulin sensitivity in the heart (see review in ref. 26). The glucose uptake in patients with type 2 diabetes has either little or no difference when compared with nondiabetic control subjects^27^,^28^. This is particularly evident when plasma FFA levels are matched^29^. These findings support two different ideas: 1) the heart may be less susceptible to insulin resistance; 2) the constant exposure of the heart to high FFA and glucose could exert toxic effects. It is thus interesting to test whether the observed Insulin-Inpp5f-Akt feedback loop is specific in heart. In addition, the increase of Inpp5f by FFA and high glucose also provides molecular evidence how high FFA and glucose exert toxic effects in cardiomyocytes.

Decreased glucose uptake/oxidation and increased fatty acids uptake/oxidation in the heart is a metabolic trait of diabetic heart, which in turn exerts toxic effects by blunting Akt activity.The decrease in insulin signaling by increased FFA uptake and oxidation is explained to come from at least 2 molecular events^30^. First, FFA-mediated ceramide accumulation seems to induce atypical PKCζ-dependent inhibition of PKB/Akt^31^. Secondly, FFA-dependent diacylglycerol accumulation induces conventional and novel PKCs activation that, in turn, could provoke the serine phosphorylation of IRS1, thereby inhibiting the PIK3/PKB/Akt pathway^32^,^33^. Our study here provides the third explanation: FFA increases Inpp5f through NF-κB, which in turn inhibits PIK3/PKB/Akt pathway and thus impedes insulin sensitivity (Fig. 7D).

Notably, we here observed an overt variation of Inpp5f expression and cardiac function among individuals in the same group, which seems different from previous studies. One explanation is that the metabolism is sensitive to the environment and mouse status, and the metabolism variation among different mice augments the difference of Inpp5f due to its instinct complex regulation. Our studies here provide a good example how variation occurs in the metabolism study. It is important to note that our findings are mainly based on the correlation study in mouse model and mechanism study in the cell culture model. It would be necessary to clarify the function of Inpp5f in diabetes by using Inpp5f transgenic and knockout mice. And it is also to test whether the findings also hold true in human by collecting clinical data.

It is also important to note that there were no significant differences of the Inpp5f expression levels in STZ-induced diabetic mice, although the cardiac functions (systolic and diastolic) were different. The apparent inconsistence might stem from the complicated regulation of Inpp5f by both insulin and blood glucose/lipid levels. In the control mice, Inpp5f could be increased by high insulin and decreased by low blood glucose/lipid levels. In contrast, in the STZ mice, Inpp5f is upregulated by hyperglycemia and hyperlipidemia and decreased by low insulin signal. What’s more, in the control mice, insulin signal should be the dominant regulator of Inpp5f expression, and Inpp5f expression in such a context keeps AKT signal in a physiological level via the feedback loop mechanism. Such a feedback loop might explain that no correlation between cardiac function and Inpp5f expression in the control group. While in the STZ induced diabetic mice, where insulin signal is deficient, Inpp5f is mainly regulated hyperglycemia and hyperlipidemia. Inpp5f expression correlates well with hyperglycemia and hyperlipidemia in the context of diabetes, and thus inversely correlates with cardiac function.

In summary, our study has revealed that Inpp5f provides as a negative feedback regulator of insulin signaling. Increase of Inpp5f in diabetes due to hyperglycemia and hyperlipidemia plays an important role in diabetic cardiomyopathy. Therapeutic reducing the increased Inpp5f will be a promising way for the treatment of diabetic cardiomyopathy.

## Materials and Methods

### Induction of Diabetes and intervention with aspirin

All experiments involving animals were performed in adherence with the Guide for the Care and Use of Laboratory Animals, and approved by the Fourth Military Medical University Committee on Animal Care. For the STZ diabetic model, male C57BL/6 mice at the age of 6–8 weeks were used. They were randomly grouped into normal and diabetic mice as they received 5 days of daily (i.p.) injection of vehicle (0.1 mol/l citrate buffer, pH4.5) alone or streptozotocin (STZ; 50 mg/kg body weight, Sigma, USA). For the HFD induced diabetic mouse model, C57BL/6 mice were maintained on a HFD (60% fat) for 26 weeks. Controls were fed standard rodent food (chow) for the same duration.

To test whether aspirin, an inhibitor of inflammation could improve the cardiac function, HFD induced diabetic mice were randomly divided into 2 groups (n = 7 each) receiving either placebo or aspirin (40 mg/L) in their drinking water. The drinking water was replaced with fresh water every other day.

### H9C2 cell culture and treatments

H9C2 cells, a permanent cell line from embryonic rat heart tissue, were originally purchased from American Type Culture Collection (ATCC, USA). The cells were cultured in Dulbecco’s modified Eagle’s medium (Hyclone, USA) supplemented with 10% fetal bovine serum (Hyclone, USA) and antibiotics (Invitrogen, USA). The medium was replaced every 2–3 days. H9C2 cells grown under normal glucose concentration (8.3 mM) were used as basal control. For high glucose treatment, H9C2 cells were treated with glucose at the final concentration of 25 mM. For the FFA treatment, H9C2 cells were added with Palmitate: BSA solution, with the Palmitate final concentration of 0.5 mM. Insulin (Sigma, USA) at the concentration of 10 nM or otherwise indicated were added for indicated time for different analysis. For Sp1 inhibition, H9C2 cells were pretreated with 100 nM Mithramycin A (cat# sc-200909, Santa Cruz, USA) 2 hrs before insulin addition. For NF- κB inhibition, H9C2 cells were pretreated with 10 μM Bay 11-708 (cat# B5556, Sigma, USA) 2 hrs before insulin addition.

### Western blot analysis

The control and diabetic hearts and H9C2 cells were harvested and lysed by RIPA containing a protease inhibitor cocktail (Roche, Germany). Proteins were subjected to SDS-PAGE gels, and transferred to nitrocellulose membranes (Millipore, USA). After blocking with 5% non-fat milk, the membranes were probed overnight at 4 °C with Inpp5f (1:500, cat# SAB2700848, Sigma, USA), p-Akt Ser473 (1:1000, cat# 9271, Cell signaling, USA), total Akt (1:1000, cat# 9272, Cell signaling, USA), p65 (1:1000, ab32536, Abcam, USA), Sp1 (1:1000, cat# 5931, Cell signaling, USA), β-actin (1:1000, cat# AB10024, Sangon, China), Lamin B (1:500, cat# BA1228, Boster, China) antibodies. Secondary antibodies conjugated to IRDye TM 800 (1:15 000, Rockland, USA) were detected using an Odyssey infrared imaging system (LI-COR, USA).

### Quantitative real-time PCR

Mouse tissues were harvested and frozen immediately in liquid nitrogen and stored at −80 °C until use. cDNA was synthesized with QuantiTect Reverse Transcription Kit (Takara, China). Expression analysis of the reported genes was performed on BIO-RAD CFX96 with the Power SYBR Green PCR Master Mix (Takara, China). β-Actin was used to normalize sample amplification. Primers used are listed in Supplementary Table S1 online.

### Cardiac Histology

All tissues were fixed in 4% paraformaldehyde and ten transferred to 1× PBS, followed by paraffin embedding. Sections of 4 μm were used for HE and Masson staining.

### Flow cytometry

Cells with either si-NC or si-Inpp5f transfection were placed onto 6 well plate and changed with fresh medium before adding the fluorescent 2-NBDG (cat# N13195, Invitrogen) or 2-NBDG together with insulin. Plates were incubated at 37 °C with 5% CO2 for 30 min before flow cytometry analysis. The 2-NBDG uptake reaction was stopped by removing the incubation medium and washing the cells twice with pre-cold phosphate buffered saline (PBS) before flow cytometry using a FACScalibur (Becton Dickinson Immunocytometry Systems).

### Luciferase reporter assay

Mouse Inpp5f promoter region and the first exon was amplified and cloned into pGL3 basic promoter using primers listed in Table S1. Mutation of the putative NF-κB binding site was done using the Multipoints mutagenesis kit per the manual instruction (Cat# R407, Takara, China). For luciferase assay, 400 ng Inpp5f promoter reporter or the mutant counterpart was co-transfected with the internal control pRL-TK into H9C2 cells. Cells were further treated as indicated. After transfection for 48 hours, H9C2 cells were lysed using passive lysis buffer. Firefly and Renillar luciferase activities were analyzed using the dual-luciferase reagent assay kit (Promega) according to the manufacturer’s instructions.

### Inpp5f overexpression and knockdown

To overexpression of Inpp5f in H9C2 cells, the full-length of Inpp5f open reading frame (3396 bp) was cloned into the Adenovirus vector GV314 (Genechem, China) in frame fusion with 3× Flag, which is subsequently packaged into adenovirus. H9C2 cells were infected with either the control or the Inpp5f expressing adenovirus at the MOI = 100. For knockdown of Inpp5f in H9C2 cells, specific RNAi duplex targeting rat H9C2 cells and the scramble RNAi control was synthesized in Genepharma (China) and transfected using Lipofectamine 2000 at the concentration of 100 nM.

### ChIP assay

H9C2 cells with indicated insulin, high glucose or FFA treatment was harvested for ChIP analysis. Briefly, formaldehyde cross-linked proliferating H9C2 cells (1 × 10^7^ cells/sample) were quenched with ice-cold 0.125 M glycine before subject to ChIP. Cells were first treated with hypotonic buffer and then the nucleic were pelleted and resuspended in 5 ml RIPA buffer containing Protease/Phosphatase Inhibitor Cocktail, and sonicated to shear the cross-linked DNA to an optimal fragment size ranging from 200 to 1000 bp. 5% of the sonicated samples were collected as Input and the rest were subjected to next steps. The sonicated samples were incubated with 1 μg control IgG and anti-Sp1 or anti-p65 antibodies to immunoprecipitate the p65 and Sp1 bound DNA fragment. The bound DNA was analyzed by PCR analysis. ChIP-PCR primers were listed in Supplementary Table 1.

### Blood biochemistry and tissue Akt activity

Serum insulin assays were performed using a standard ELISA kit (cat# EZRMI-13K, Millipore, USA) per the manufacturer’s instructions. Tissue Akt activity was measured from tissue lysates using the Akt Kinase Activity Assay Kit (ab139436, Abcam, USA) as manually instructed. Blood glucose, triglyceride, and cholesterol were examined by Abbott Auto-analyzer (Ci8200).

### Echocardiographic examination

Echocardiograms were performed on conscious, gently restrained mice using Esaote Twice System with the SL3116 transducer. LVEDD and LVESD were measured from M-mode recordings. Ejection fraction value was calculated as (LVEDD-LVESD)/LVEDD and expressed as a percentage. Measurements of diastolic dysfunction, as seen from isovolumetric relaxation time was made from 2D parasternal short axis views in diastole.

### Visualization of Nanoparticle Uptake *in Vivo*

For delivery of siRNAs into the heart, siRNAs were conjugated with 10 nm gold nanoparticles as described previously^34^. AuNP loaded with siRNAs were injected via tail vein. For visualization of the uptake of siRNAs, mice were injected with Cy3 labeled AuNP and 24 hours later the mice were sacrificed and the hearts were harvested and sectioned in OCT. The uptake of nanoparticles was visualized under FV-1000/ES confocal microscope (Olympus, Tokyo, Japan).

### Statistics analysis

The statistical analyses were performed using GraphPad Prism software (Graphpad, San Diego, CA). For experiments with more than 2 groups, the differences between groups were compared by a one-way analysis of variance (ANOVA) followed by Dunnett’s test, in which all groups were tested against a control group as a reference. For experiments with only 2 groups, the unpaired Student’s *t*-test was used for comparisons of group means. Linear regression also used for statistical analysis. P-values <0.05 were considered to represent significant differences.

## Additional Information

**How to cite this article**: Bai, D. *et al*. Hyperglycemia and hyperlipidemia blunts the Insulin-Inpp5f negative feedback loop in the diabetic heart. *Sci. Rep*. **6**, 22068; doi: 10.1038/srep22068 (2016).

## Supplementary Material

Supplementary Information
