# Supplementary material for: Hyperglycemia and hyperlipidemia blunts the Insulin-Inpp5f negative feedback loop in the diabetic heart
Source: Sci Rep. 2016 Feb 24;6:22068. doi: 10.1038/srep22068 (PMC4764951; doi:10.1038/srep22068)
Supplement: Supplementary Information [file srep22068-s1.pdf]

Hyperglycemia and hyperlipidemia blunts the Insulin-Inpp5f negative feedback loop in the diabetic heart

Danna Bai[1, 2a], Yajun Zhang[3a], Mingzhi Shen[1, 4a], Yongfeng Sun[2], Qing Xia[2], Yingmei Zhang[1], Xuedong Liu[5*], Haichang Wang[1*], Lijun Yuan[3*]

1. Department of Cardiology, Xijing Hospital, the Fourth Military Medical University, Xi'an 710032, China

2. 323 Hospital of PLA, Xi'an 710054, China

3. Department of Ultrasound Diagnostics, Tangdu Hospital, the Fourth Military Medical University, Xi'an 710038, China

4. Department of Cardiology, Hainan Branch of PLA General Hospital, Sanya 572013, China

5. Department of Neurology, Xijing Hospital, the Fourth Military Medical University, Xi'an 710032, China

[a] *These authors (Danna Bai, Yajun Zhang and Mingzhi Shen) contribute equally to this work.*

[*] *Co-corresponding author:* Lijun Yuan, Department of Ultrasound Diagnostics, Tangdu Hospital, the Fourth Military Medical University, Xi'an 710038, China
Tel: 86-29-84777171; Fax: 86-29-84777471. Email: yuanlj@fmmu.edu.cn.

[*] *Co-corresponding author:* Haichang Wang, Department of Cardiology, Xijing Hospital, the Fourth Military Medical University, Xi'an 710032, China
Tel: 86-29-84775183; Fax: 86-29-84771024; Email: wanghc@fmmu.edu.cn.

[*] *Co-corresponding author:* Xuedong Liu, Department of Neurology, Xijing Hospital, the Fourth Military Medical University, Xi'an 710032, China
Tel: 86-29-84771056; Fax: 86-29-84771056; Email: liuxued@fmmu.edu.cn

Figure S1 Cardiac expression dynamic of Inpp5f and its relation with blood biochemistry in STZ induced diabetic mice.

(A-D) Serum insulin (A), blood glucose (B), cholesterol (C), and triglyceride levels in mice treated with STZ were recorded at the indicated time. $^{*}P < 0.05$ versus day 0, n=6.

(E) mRNA expression of Inpp5f in above samples were analyzed by qPCR and normalized to Gapdh. $^{*}P < 0.05$; versus respective control group.

(F) Representative data of immunohistochemistry staining of Inpp5f in the hearts from control mice (a), STZ treated mice at day 45 (b) and day 60 (c).(G) Hearts were collected at indicated time after STZ treatment. Expression of Inpp5f at protein level was detected by western blot and compared with day 0. (ctrl, n=2; STZ, n=3 at each time point)

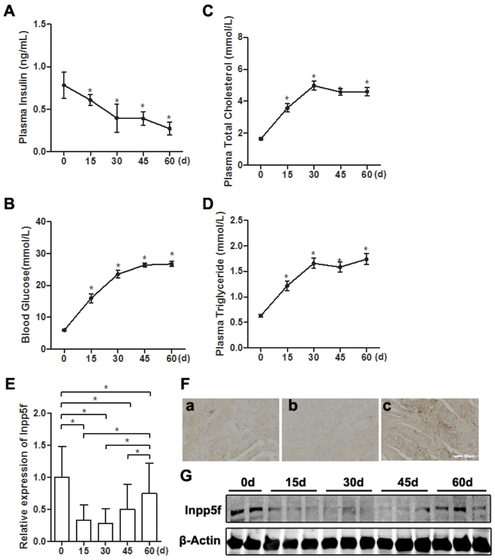
A
Plasma Insulin (ng/mL)
C
Plasma Total Cholesterol (mmol/L)
B
Blood Glucose(mmol/L)
D
Plasma Triglyceride (mmol/L)
E
Relative expression of Inpp5f
F
G
0d
15d
30d
45d
60d
Inpp5f
β-Actin

Fig. S1

Figure S2 Increased Inpp5f in STZ induced diabetic hearts predicts severe cardiac dysfunction.

(A) HE staining of the hearts from Ctrl and STZ treated mice 60 days after induction. Data presented are a representative of 6 mice in each group.

(B) M-mode echocardiography of Ctrl and STZ treated mice 60 days after induction. Representative data of 6 mice in each group.

(C) Representative data of Masson Trichrome staining of the hearts from Ctrl (a) and STZ (b) fed mice.

(D) Ejection fraction (EF) value of cardiac systolic function in both Ctrl and STZ groups. *$P < 0.05$, n=6.

(E) Correlation of Inpp5f expression and EF value in all the 6 control and 6 STZ treated mice. The lowest expression of Inpp5f was set as 1.

(F) Correlation of Inpp5f expression and EF value in 6 STZ induced diabetic mice only.

(G) Isovolumic relaxation time (IVRT) of cardiac diastolic function in both Ctrl and STZ groups. *$P < 0.05$, n=6 in each group.

(H) Correlation of Inpp5f expression and IVRT value in all the 6 control and 6 STZ treated mice. *$P < 0.05$.

(I) Correlation of Inpp5f expression and IVRT value in 6 STZ induced diabetic mice only. *$P < 0.05$.

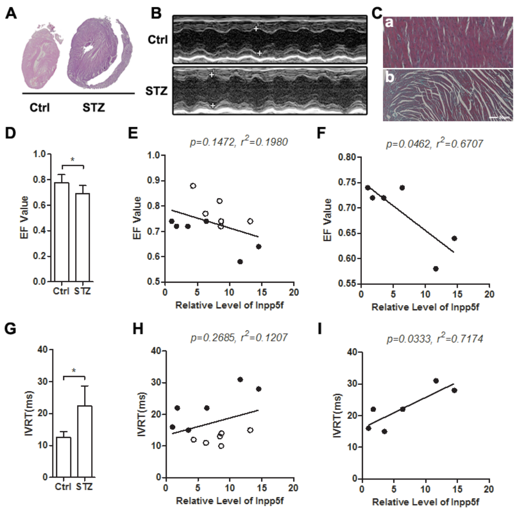

Fig. S2

Figure S3 Correlation between Inpp5f expression and Akt activity in STZ induced diabetic hearts. Inpp5f expression level and relative Akt activity in STZ induced diabetic hearts were analyzed. n=6

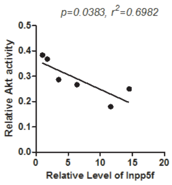
p=0.0383, r2=0.6982
Relative Akt activity
0.5
0.4
0.3
0.2
0.1
0.0
0
5
10
15
20
Relative Level of Inpp5f

Fig. S3

Figure S4 AuNP mediated delivery of siRNA into the heart. Cy3 labeled siRNAs were conjugated with the gold nanoparticles and injected via tail vein. Distribution of the siRNAs in the heart was analyzed by fluorescent microscope. (Scale bar=50 μ m)

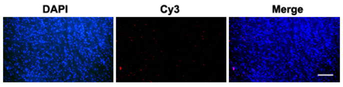

Fig. S4

Figure S5 Alignment of Inpp5f promoter and partial sequence of the first exon till ATG across human, mouse and rat. High conservation of the regulatory region of Inpp5f is observed, with about 87% identity between mouse and rat. The NF-κB binding sites are labeled with red box, while the Sp1 binding sites are labeled with black box.

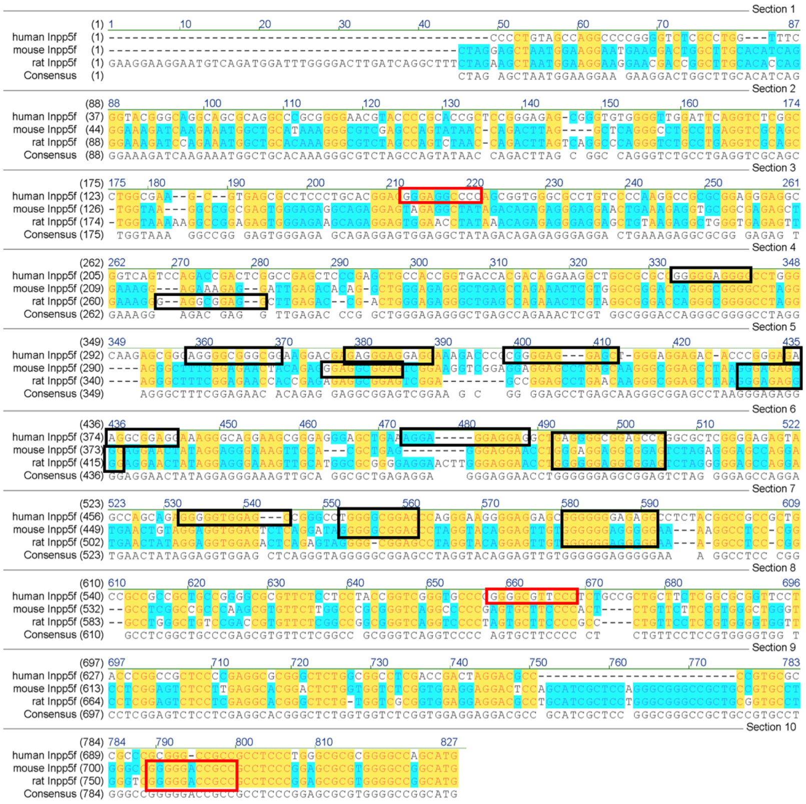

Section 1
Section 2
Section 3
Section 4
Section 5
Section 6
Section 7
Section 8
Section 9
Section 10
human Inpp5f
mouse Inpp5f
rat Inpp5f
Consensus

Fig. S5

Table S1 Primers and sequence used in this study

Name	Sequence
Mouse Inpp5f qPCR	5’-GATGACATTCACCCACGAT-3’
	5’-GTCTCCACATAATTCGCAAC-3’
Rat Inpp5f qPCR	5’-GGAGGCCACTTGTGTAGAT-3’
	5’-TTTTATCCACTCCTCTTCG-3’
β-actin qPCR (common for both mouse and rat)	5’-AGAGGGAAATCGTGCGTGAC-3’
	5’-TTCTCCAGGGAGGAAGAGGAT-3’
SiNC	5’-UUCUCCGAACGUGUCACGUTT-3’
Si-Inpp5f-1	5’-GGAGUUAACUCAGCAAAUATT-3’
Si-Inpp5f-2	5’-GGCUAACUCAUGGGAUAAATT-3’
Inpp5f promoter cloning	5’- GGAAGCTTGCTAATGGAAGGAATGAAGG -3’
	5’- GGCCATGGCGGCCCCACGCGCTCCGG -3’
Mutant NFκB binding site	5’-CCTGGGCC*GATAGACATAC*GCCTCCCG-3’
ChIP primer (Sp1)	5’-TCGGAGCCGGAGCCTGAACAA-3’
	5’-CTCCACCTCCTATAGTTCAT-3’
ChIP primer (NFκB)	5’-TGGAGGAGGACGCCTGCAT-3’
	5’-TAGTGGTCCTTGGCCTGAA -3’
